# Comprehensive review of the evidence regarding the effectiveness of community–based primary health care in improving maternal, neonatal and child health: 6. strategies used by effective projects

**DOI:** 10.7189/jogh.07.010906

**Published:** 2017-06

**Authors:** Henry B Perry, Emma Sacks, Meike Schleiff, Richard Kumapley, Sundeep Gupta, Bahie M Rassekh, Paul A Freeman

**Affiliations:** 1 Department of International Health, Johns Hopkins Bloomberg School of Public Health, Baltimore, Maryland, USA; 2 UNICEF, New York, New York, USA; 3 Medical epidemiologist, Lusaka, Zambia; 4 The World Bank, Washington, District of Columbia, USA; 5 Independent consultant, Seattle, Washington, USA; 6 Department of Global Health, University of Washington, Seattle, Washington, USA

## Abstract

**Background:**

As part of our review of the evidence of the effectiveness of community–based primary health care (CBPHC) in improving maternal, neonatal and child health (MNCH), we summarize here the common delivery strategies of projects, programs and field research studies (collectively referred to as projects) that have demonstrated effectiveness in improving child mortality. Other articles in this series address specifically the effects of CBPHC on improving MNCH, while this paper explores the specific strategies used.

**Methods:**

We screened 12 166 published reports in PubMed of community–based approaches to improving maternal, neonatal and child health in high–mortality, resource–constrained settings from 1950–2015. A total of 700 assessments, including 148 reports from other publicly available sources (mostly unpublished evaluation reports and books) met the criteria for inclusion and were reviewed using a data extraction form. Here we identify and categorize key strategies used in project implementation.

**Results:**

Six categories of strategies for program implementation were identified, all of which required working in partnership with communities and health systems: (a) program design and evaluation, (b) community collaboration, (c) education for community–level staff, volunteers, beneficiaries and community members, (d) health systems strengthening, (e) use of community–level workers, and (f) intervention delivery. Four specific strategies for intervention delivery were identified: (a) recognition, referral, and (when possible) treatment of serious childhood illness by mothers and/or trained community agents, (b) routine systematic visitation of all homes, (c) facilitator–led participatory women’s groups, and (d) health service provision at outreach sites by mobile health teams.

**Conclusions:**

The strategies identified here provide useful starting points for program design in strengthening the effectiveness of CBPHC for improving MNCH.

In recent decades, much of the funding for global health has concentrated on technical cooperation pertaining to strengthening narrowly focused vertical programs, such as control of HIV, malaria and tuberculosis, and expanding immunization coverage. However, in order to accelerate progress in the reduction of readily preventable deaths of children and mothers, there have been calls for more direct funding for integrated maternal and child health programs [1], health systems strengthening [2], integration of key interventions via a continuum of care [3,4], and stronger community participation [5]. However, none of these calls have sufficiently emphasized the importance of strengthening community–based service delivery strategies for accelerating progress by achieving high levels of coverage of evidence–based interventions. Too often, attention has been focused on the technical aspects of interventions rather than on the strategies and support systems that are needed to achieve high levels of population coverage.

Previous reviews have highlighted family and community practices that are important for maternal, newborn and child health [6] as well as specific technical interventions that can be provided in communities [7–10], but none have to date focused specifically on the implementation strategies that effective projects have used. This paper summarizes the various approaches used by the programs, projects and studies (hereafter referred to as projects) whose effectiveness has been assessed and included in a comprehensive database.

## METHODS

We conducted a comprehensive review of the effectiveness of community–based primary health care (CBPHC) in improving maternal, newborn and child health (MNCH) by reviewing 12 186 published reports of community–based programs for improving MNCH in low– and middle–income countries. 552 of these reports qualified. An additional 148 reports were identified from the “grey” literature (documents publicly available on the internet) and books. A total of 700 assessments were included in this review. A full description of the search strategy and creation of the database is available elsewhere [11].

Of particular importance for this paper is that a data extraction form was designed to capture as much information as possible in the document containing the project’s assessment that describes the project strategies and what role the community played. We did not attempt to force any strict definition of the term “community” in the analysis of the findings since there was no uniform definition used in the projects or by the reviewers. By strategies we mean the activities that these projects used to make the intervention effective – to plan the project, engage partners (including the community), implement the project, engage in associated activities not directly related to intervention delivery, and evaluate the project. The data extraction forms used to collect information from the assessments were designed to capture the available information regarding strategies used for project implementation. In particular, open–ended descriptions of project implementation were completed by reviewers.

A copy of the data extraction form is contained in Online Supplementary Document of the above–mentioned paper [11]. The form allows for open–ended as well as close–ended responses related to strategies and community engagement. Data were extracted from each assessment by two independent reviews and a third reviewer resolved any differences between the first two reviews.

The maternal, neonatal and child health database was searched carefully to identify all information that described the strategies that were used by projects. All available evidence in the database regarding strategies for project implementation was reviewed by reviewing all the open–responses individually and summarizing common themes as well as by adding up the number of responses to close–ended questions.

### RESULTS

We identified six categories of strategies used by the projects in our database: (a) program design and evaluation, (b) community collaboration, (c) education for community–level staff, volunteers, beneficiaries and community members, (d) health systems strengthening, (e) use of community–level volunteers and workers (hereafter referred to as community health workers, or CHWs), and (f) intervention delivery. **Table 1** summarizes these strategies. The strategies were not mutually exclusive and most projects used at least several of these strategies and, in fact, some of the strategies fit into several categories (eg, participatory women’s groups).

**Table 1:** Summary of strategies used by CBPHC projects to improve child health

Category of strategy	Specific strategy
**Program design and evaluation**	Knowledge, practice and coverage (KPC) household surveys
	Participatory Rural Appraisal (PRA)
	Village rosters of beneficiaries
	Census–taking
	Disease surveillance (based on information provided by community–based workers and communities)
	Prospective registration of vital events (pregnancies, births and deaths)
	Retrospective mortality assessment (based on maternal birth histories)
	Determination of cause of death from verbal autopsies
	Engagement of communities in planning and evaluation
**Community engagement**	Collaboration with or formation of village health committees and/or collaboration with local leaders
	Formation and/or support of women’s groups
	Sharing locally obtained health–related data with the community
	Participatory Rural Appraisal (PRA)
	Formation and/or support of microcredit programs for women
	Involvement of older family members (men and grandparents/mothers–in–law)
**Education of community–level staff, volunteers, beneficiaries and community members in general**	Social marketing (media campaigns, posters, radio, etc.)
	Skits, stories and games for health education messages
	Peer–to–peer education (volunteer mothers visiting neighbors with targeted health messages)
	Education of grandmothers
	Positive deviance inquiry
	Training of trainers/cascade training
**Health systems strengthening**	Identification of cases of childhood illness in need of referral
	Strengthening referral system
	Strengthening of quality of care at referral facility
	Strengthening of supervisory system
	Strengthening logistics/drug supply system
	Training of providers at primary health center
	Training of community–level health care providers
**Use of community health workers**	Intermittent use of minimally trained volunteers for highly specific, targeted activities
	Use of volunteers for regular ongoing activities
	Use of trained and paid workers with 1–11 months of training
	Use of trained and paid workers with 1 year of training
**Intervention delivery**	Community case management
	Home visits
	Participatory women’s groups
	Provision of health services at community outreach points by mobile teams from peripheral facilities

### Strategies for program design and evaluation

Strategies for project design and evaluation shown in **Table 1** often included baseline and endline knowledge, practice and coverage (KPC) population–based household surveys. These made it possible to measure changes in intervention coverage in the program population as well as changes in childhood nutritional status as determined by anthropometry. Oftentimes, community members served as interviewers or collaborators for these surveys. In some projects, Participatory Rural Appraisal (PRA), an approach that incorporates the viewpoints of local people in the planning and management of development projects, was used to guide project planning or evaluation.

Various approaches were used to determine the beneficiary population (usually mothers, including pregnant women, and their young children) such as household censuses carried out by the project in collaboration with community members or the development of village rosters of beneficiaries. Sometimes projects included a disease–surveillance component using information provided by community–based workers and communities. Examples are surveillance for acute flaccid paralysis (to identify possible cases of polio) and for other vaccine–preventable diseases such as neonatal tetanus and measles. Some projects measured changes in mortality directly, either through prospective vital events registration as in Care Group projects [12] and in the pioneering CBPHC field project at Gadchiroli, India, conducted by SEARCH [13,14] or through retrospective measurements obtained from maternal birth histories [15,16]. Verbal autopsy methods have been used to assess the leading causes of child deaths in the project area and whether or not the cause of death “structure” has changed over time [17]. Finally, communities have been consulted during the project planning phase as well as at the time of project evaluation. In these circumstances, community members assist with data collection for structured surveys and participate as key informants or participants in focus group discussions.

### Strategies for community engagement

Community engagement takes many forms and is commonly mentioned in the assessments included in our database (**Table 1**). Village health committees are often formed if they were not previously in existence, and projects work with them in project design, implementation and evaluation. Community leaders, including local religious leaders, are commonly consulted. Communities are often mobilized to participate in health campaigns or to practice key healthy behaviors. Many projects have worked with existing community groups or formed new ones, often women’s groups. Activities that empower women are common forms of community engagement, including education and consciousness raising of women as well as formation and support of women’s microcredit and savings groups.

Communities are commonly requested to participate in the selection of CHWs and to provide support to them and participate in their supervision. Finally, in some projects, special activities are geared toward engaging fathers, mothers–in–law, traditional healers and local drug sellers. Finally, though not commonly, projects have engaged communities by sharing surveillance and evaluation results. Noteworthy examples of projects with strong community engagement strategies include mobilization of churches in Mozambique [12] and Nigeria [18] and national mobilization of communities and short–term community workers for national health weeks in Sierra Leone [19].

### Strategies for education of community–level staff, volunteers, beneficiaries and community members in general

Assessments of the effectiveness of projects included in our database have adopted many innovative approaches to educating CHWs, beneficiaries, and community members as a whole. Some have used social marketing channels such as radio and posters to convey key messages to the entire community. Others have conveyed health education messages through skits, puppet shows and games that engaged children, mothers, or the entire community. One noteworthy example of this approach is the World Relief child survival project in Cambodia [20,21].

Other approaches involved teaching health education messages to volunteer or paid community workers (who most often are mothers) who then conveyed them to their neighbors at the time of home visits or at meetings of small groups of neighbors. Sometimes projects targeted grandmothers for health education messages since they are respected and influential elders in the community. One particularly innovative educational strategy used in some projects is positive deviance inquiry, usually for addressing childhood undernutrition [22]. With this strategy, mothers of undernourished children in a village learn from the mothers of well–nourished children in the village how they care for their children – not just how they feed them but how they care for them more broadly.

Another approach used by some projects is called Care Groups [23], which involves training a small number of master trainers in a project area with a set of health education messages. These trainers each then train another set of trainers who then train another set. Through this “cascade training” approach, large numbers of peer–to–peer counselors can be trained to convey key messages to every household.

### Strategies for health systems strengthening

Many CBPHC projects carried out health system strengthening activities of various sorts. One of the most common was providing mothers and their families with educational messages about warning signs for serious childhood illness or about pregnancy and childbirth for which care should be sought at a health facility. A stronger health system is one in which people seek care appropriately and, when potentially serious conditions are present, prompt care is sought. This is core feature of the approach known as Community–based Integrated Management of Childhood Illness (C–IMCI), utilized in many child survival projects funded by the US Agency for International Development, often with marked expansions of geographic coverage of key child survival interventions. A publication highlighting a number of these projects has been published [24].

Another approach has been to work with communities to establish emergency transport systems to ensure that mothers and children can access the nearest health facility whenever a complication arises and also ensure that the family can obtain transport at a fixed, fair, and affordable price. These referral systems are sometimes linked to insurance schemes whereby families pay small amounts of money on a regular basis, usually during pregnancy, to cover all or most of the cost of such transport if needed. One such approach has been developed by Curamericas for isolated mountainous communities in Guatemala [25,26].

Many projects, while implementing community–based interventions, also engage in activities to strengthen the quality of care provided at primary health care centers or referral hospitals, including the capacity of facilities to accept and care for referrals. This often takes the form of training staff who work there or helping the facility to improve its own stock of drugs and supplies.

Other approaches include improving the quality of the community–based health system itself by providing training to CHWs, by strengthening the supervision provided to CHWs, or strengthening the logistics/drug supply system for CHWs.

### Strategies for use of community health workers

Community–based programs often rely on various types of CHWs – trained volunteers or more formally trained and paid workers who can implement specific interventions aimed at improving MNCH. The projects in our database engaged a broad variety of CHWs. For some projects, the training lasted only a few hours or days while for others CHWs had one year or more of full–time formal training. Some CHWs received only a “per diem” payment for attending a training course or a certificate for their service, while others were formally paid government employees. Some CHWs were volunteers or workers who had been engaged for a specific local project or study while others were part of a national government–run program.

**Table 2** provides a listing and description of the types of CHWs described by reports in our database.

**Table 2:** Specific examples of community health workers (CHWs) utilized in community–based primary health care (CBPHC) projects with evidence of effectiveness in improving neonatal and child health

Category of CHW	Names given to CHWs in this category	Comment
Intermittent use of minimally trained unsalaried workers for highly specific, targeted activities	Child Health Day volunteer	May receive a per diem payment
Use of unsalaried workers for regular ongoing activities	Promoters, peer educators, malaria or nutrition agents, Care Group volunteers, animators, community case management workers, nutrition counselor mothers, bridge–to–health teams, family health workers, community surveillance volunteers, female community health volunteers	May receive certain incentives such as uniforms, per diem payment for training, or an occasional small stipend
Use of workers with 1–11 months of training who receive a salary	Health agents, community health agents, family planning agents, health surveillance assistants, *accompagnateurs,* lead mothers, *soccoristas,* Care Group facilitators (animators or promoters)	
Use of workers with 1 year or more of training who are salaried	Auxiliary nurses, community health officers, health extension workers	

### Strategies for implementation of interventions

Four types of strategies for implementing interventions were: (1) recognition, referral, and (in certain circumstances) treatment of serious childhood illness by mothers and/or CHWs; (2) routine systematic visitation of all homes, (3) facilitator–led participatory women’s groups; and (4) provision of health services at community outreach points by mobile teams from peripheral facilities.

### Community case management: recognition, referral, and (when possible) treatment of serious childhood illness by mothers and/or trained community agents

The review identified considerable evidence regarding the effectiveness of training and supervising CHWs to teach pregnant women and their families about danger signs during pregnancy and childbirth, during the newborn period, and among sick children [27–29]. CHWs can learn to recognize danger signs and they can teach these to mothers, other caregivers, and family members.

Some projects that were effective in improving neonatal and child health also trained and supported CHWs to manage these conditions themselves (or in some cases these CHWs also taught mothers how to treat these conditions). This requires, in addition to proper training, appropriate supervision and logistical support for medications and other supplies [30–33]. The community–based treatment modalities included administration of oral (and in a few cases intramuscular) antibiotics [34], administration of oral rehydration fluids, provision of highly nutritious foods available locally or commercially prepared (known as ready–to–use therapeutic foods, or RUTF), and in some cases provision of micronutrients such as iron, vitamin A and zinc. When community–level workers did not have the capacity to treat children with acute illness, they informed mothers and caretakers that urgent treatment at a referral health facility was needed. A comprehensive manual for community–based diagnosis and treatment of serious childhood illness is available for general use [35]. Integrated community case management (iCCM) for childhood illness is now being scaled up in many countries [36].

### Routine systematic visitation of homes

Routine systematic visitation of homes makes it possible to identify those in need of basic services and to provide everyone in the program population with essential health education and selected key services, particularly during pregnancy and the early neonatal period. Community–level workers who make home visits are generally able to identify pregnant women and mothers of young children, provide education to them and other family members (especially husbands and mothers–in–law), recognize danger signs during pregnancy and childhood illness, encourage referral when danger signs are present, and provide treatment for certain conditions that can be identified at the time of home visits such as growth faltering, diarrhea, pneumonia, and malaria.

Based on current evidence, the World Health Organization and UNICEF recommend that all pregnant women receive two home visits during the prenatal period, one home visit during the first 24 hours after birth, and at least one visit as soon as possible after delivery [37]. Activities that should take place during these visits include the following: education about proper nutrition, promotion of antenatal care, education about danger signs during pregnancy and childbirth, promotion of breastfeeding immediately after birth, prevention of hypothermia, and measurement of the weight of newborns to identify low–birth–weight newborns who need additional home visits. A number of studies have highlighted the difficulties many women face in accessing health facilities due to distance and cost [38]. Home visitation provides an alternative for those without ready access to health facilities.

Home visitation is also an effective means of providing counseling about breastfeeding and appropriate complementary feeding, hand washing, prevention and treatment of diarrhea, detection and treatment of childhood pneumonia, and family planning services. There are a number of variations of home visitation strategies using community–level workers, from weekly home visits for providing micronutrients to children [39] to regular monthly visitation of all homes in a program population as part of a more comprehensive approach to delivering basic services to the entire population [40].

Finally, an ongoing program of home visitation provides a foundation of trust and awareness. When children develop signs of serious illness that can be managed by CHWs (such as for pneumonia, diarrhea or malaria), families will be more predisposed to contact the CHW for early and prompt treatment.

### Participatory women’s groups

Participatory women’s groups are led by facilitators with less than two weeks of training who provide the opportunity for further empowerment and education about healthy behaviors, danger signs of serious illness, and proper care of the newborn. These groups may also address issues outside of the health domain that are a priority to the community and that may also have an indirect effect on health (such as income generation activities). These groups may also provide a vehicle for counseling about breastfeeding, birth spacing, infant feeding, hand washing, prevention and treatment of diarrhea, signs of childhood pneumonia, and danger signs during pregnancy and childbirth. Participatory women’s groups also can be effective for assisting mothers to rehabilitate malnourished children detected through growth monitoring.

The literature illustrates several effective approaches to facilitating participatory women’s groups, including the use of a participatory action–learning cycle [41,42], formation of Care Groups (10–15 women volunteers who meet with a facilitator (promoter/animator) once a month to learn a key health education message to disseminate to each of the mothers in the 10–15 households surrounding each volunteer) [43,44], and education sessions led by community mobilizers [45].

### Provision of health services at community outreach points by mobile teams from peripheral facilities

Provision of services at satellite clinics, including holding outreach immunization sessions, by mobile teams based at health centers is a common means of community–based outreach. These mobile teams may have a vehicle or more likely a motorcycle, bicycle, horse or donkey, or they may even travel by foot. The provision of immunization services by mobile health teams at points beyond a peripheral health facility is now well–developed in many low–income countries [46]. Other examples of services that can be provided through outreach include promotion of and provision of family planning services, basic antenatal care, testing for HIV and syphilis, distribution of insecticide–treated bed nets, distribution of medications to prevent or treat malaria, and growth monitoring to detect cases of childhood malnutrition.

One widely implemented variation of this strategy is Child Health Days (or sometimes called Child Health Weeks). Generally occurring twice a year, they usually include some combination of immunization administration, vitamin A supplementation, nutritional monitoring (and referral of malnourished children), and distribution of oral rehydration packets, water–purification tablets, or de–worming tablets [47,48]. Services are provided at peripheral outreach points separate from a health center such as at a school or community building or even under a tree, and home visits are often carried out in addition to reach those mothers and children who did not come to the outreach points. These children are often identified on the basis of previously developed household registers.

**Table 3** demonstrates which evidence–based child survival interventions can be implemented by which implementation modality. The interventions shown in **Table 3** are those which have been identified by the Lives Saved Tool (LiST) for inclusion in program plans for reducing under–5 mortality [49]. A more detailed discussion of these four intervention delivery strategies has been reported elsewhere [50].

**Table 3:** Child health interventions with strong evidence of effectiveness through community–based implementation

Technical intervention	Community–based intervention delivery strategy			
**Community case management**	**Home visits**	**Participatory women’s groups**	**Outreach services**	
Immunizations: BCG, polio, diphtheria, pertussis, tetanus, measles, Haemophilus Influenza Type b (Hib), pneumococcus, rotavirus immunizations for children; tetanus immunization for mothers and women of reproductive age		X		X	
Provision of supplemental vitamin A to children 6–59 months of age and to post–partum mothers		X		X	
Provision of preventive zinc supplements to all children 6–59 months of age		X		X	
Promotion of breastfeeding immediately after birth, exclusive breastfeeding during the first 6 months of life and continued non–exclusive breastfeeding beyond 6 months	X	X	X	X	
Promotion of appropriate complementary feeding beginning at 6 months of age	X	X	X	X	
Promotion of hygiene (including hand washing), safe water, and sanitation	X	X	X	X	
Promotion of oral rehydration therapy (ORT) for diarrhea with or without zinc supplementation	X	X	X	X	
Promotion of clean deliveries, especially where most births occur at home and hygiene is poor		X	X	X	
Detection/referral of pneumonia with or without provision of community–based treatment	X	X	X	X	
Home–based neonatal care (frequent home visits for promotion of immediate and exclusive breastfeeding, promotion of cleanliness, prevention of hypothermia, and diagnosis and treatment of neonatal sepsis by CHW)	X	X	X		
Community–based rehabilitation of children with protein–calorie undernutrition through food supplementation (including rehabilitation of children with severe acute undernutrition through ready–to–use dry therapeutic foods)	X	X	X	X	
Insecticide–treated bed nets (ITNs) in malaria–endemic areas		X	X	X	
Indoor residual spraying in malaria–endemic areas		X		X	
Detection/referral of malaria with or without provision of community–based treatment	X	X	X	X	
Intermittent preventive treatment of malaria during pregnancy (IPTp) and infancy (IPTi) in malaria–endemic areas		X		X	
Detection and treatment of syphilis in pregnant women in areas of high prevalence		X		X	
Promotion of HIV testing in pregnant women and prevention of mother–to–child transmission (PMTCT) of HIV infection	X	X	X	X	
Iodine supplementation in iodine–deficient areas where fortified salt is not consumed		X	X	X	
Provision and promotion of family planning services		X	X	X	

*Outreach of health facility staff includes holding mobile clinics and/or immunization sessions at specified locations outside of health facilities in outlying communities on a regular basis.

### Frequency of selected program–related processes

When program assessments that qualified for the review underwent data extraction, reviewers were asked to describe the degree to which communities were involved in various aspects of the project. Some of the findings are contained in **Table 4**. These findings demonstrate a high degree of community engagement, both in the maternal as well as the neonatal/child health CBPHC projects. More than three–fourths of the projects trained CHWs and more than one–third engaged communities in the formation or support of community groups as well as in the planning of project activities. 81% of the projects engaged communities in project implementation, and more than half promoted partnerships between the community and the health program, promoted the use of local resources, or promoted community empowerment. Almost half promoted women’s empowerment, one–third promoted leadership in the community, and one–quarter promoted equity. 40% of the projects involved the community in the project evaluation. These findings are highly likely to underestimate the true situation since a large portion of the assessments did not go into this level of detail in describing the community engagement component of the project. Information provided in the assessment was rarely sufficient to provide any deeper understanding of the quality of community engagement or details of how community engagement was actually carried out.

**Table 4:** Community involvement in the implementation of maternal, neonatal and child health CBPHC projects included in the database

Stage of implementation	Activity	Percentage of assessments of maternal CBPHC projects that describe activity (n = 152)	Percentage of assessments of neonatal and/or child health CBPHC projects that describe activity (n = 548)	Percentage of assessments of all maternal, neonatal and/or child health CBPHC projects combined that describe activity (n = 700)
**Inputs**	Training of CHWs	86.3	74.0	76.6
	Formation and/or support of community groups	53.6	35.5	39.5
	Community involvement in planning	46.4	36.1	38.3
**Processes**	Community involvement in implementation	90.8	78.1	80.9
	Promotion of partnerships between the community and the health program	73.2	53.6	57.8
	Promotion of the use of local resources	74.5	53.2	57.8
	Promotion of community empowerment	62.7	53.6	55.6
	Promotion of leadership in the community	41.8	30.4	32.9
	Promotion of women’s empowerment	62.7	40.6	45.4
	Promotion of equity	24.8	24.8	24.8
**Evaluation**	Community involvement in evaluation	50.3	37.5	40.3

## DISCUSSION

This analysis of strategies used by effective community–based programs for improving MNCH has documented a high degree of community engagement in project implementation. Six categories of strategies were identified: (a) program design and evaluation, (b) community collaboration, (c) education for community–level staff, volunteers, beneficiaries and community members, (d) health systems strengthening, (e) use of CHWs, and (f) intervention delivery. Within each strategy category, community engagement was an essential element for strategy implementation. By its very nature, CPBHC requires community engagement for virtually all aspects of programming. Each of these aspects of community engagement are part of the process of building capacity within the community for the benefit of the health program and its capacity to improve the health of mothers, neonates and children. Further elaboration of these strategies as they pertain specifically to maternal, neonatal and child health are discussed in other articles in this series [51–53].

**Figure 1:**
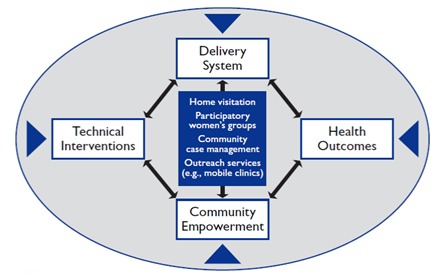
A conceptual framework for planning, implementing and evaluating community–based primary health care programs for improving maternal, neonatal and child health. Blue triangles represent contextual factors.

In general, the details of community–based strategies and approaches used by projects to improve MNCH have not been well described in the peer–reviewed scientific literature, where the focus is usually on the health impact of the intervention, or set of interventions, rather than on describing in sufficient detail the exact implementation strategies used to achieve that impact. The findings of this review provide insights into the richness of this dimension of implementation strategies and its importance for program effectiveness. **Figure 1** contains a framework that attempts to capture the importance of community empowerment for improving the health of mothers, neonates and children. The delivery process, along with the technical content of the interventions, is embedded in the eventual health outcomes produced together by the health system working with the community.

The framework in **Figure 1** and in fact the strategies identified in this article as well as the interventions identified in other articles in this series all highlight the importance of community engagement and community–based delivery of interventions outside of health facilities in order to reach those who need services. As Gwatkin et al. observed in their 1980 comprehensive review of the effectiveness of programs improving child health and nutrition [54]:

“*Unless services reach those in need, even the best–conceived primary health and nutrition care programs can obviously have little impact on mortality. Thus, … the development of plans for getting services to the people is as important as are decisions concerning which services should be offered.*”

CBPHC involves, above all, getting services to those who need them.

**Figure 1** emphasizes the importance of context. In fact, strategies in general are context– specific. In order for community–based programs to be successful, the context must be carefully considered in order to select the most appropriate combinations of interventions and implementation strategies. Program effectiveness in improving MNCH in a given geographical area requires knowing the local epidemiological priorities (ie, the most frequent and readily preventable or treatable serious conditions) as well as the feasibility of achieving high coverage of evidence–based interventions targeting the epidemiological priorities given the available resources, logistical challenges, contextual constraints (including health system constraints), and available implementation strategies.

The assessments making up our database are derived largely from small demonstration projects, short–term trials, and efficacy studies of one or a small number of interventions. More independent, rigorous assessments of large–scale integrated programs at scale carried out for five or more years are needed. There are few examples of rigorous assessments of CBPHC at scale over a longer time period. However, these few studies show that the bottlenecks to the effectiveness of large–scale programs include assuring that the number of CHWs and their supervisors is sufficient for the population being served, and that CHWs receive adequate support and supervision, including the basic commodities they need to do their work [55,56]. Future research is needed to rigorously assess the effectiveness of community–based approaches at scale in relatively routine conditions [57].

Elsewhere in this series we review the common characteristics of four projects that have long–term evidence of effectiveness [58]. A more in–depth analysis of the strategies and effectiveness of the larger projects included in our review has not been carried out. Although such an analysis would be useful, unfortunately it is beyond the scope of the current series of articles. Questions that might be addressed through such an analysis include:

- Is effectiveness weakened as projects scale up? If not, what specific steps were taken to maintain quality and effectiveness?
- What kinds of community engagement and what kinds of community–level workers were used in different projects, and how did these features contribute to effectiveness?
- What is the contribution of civil society and NGOs to larger–scale projects and how do these contributions affect the effectiveness of public–sector programs?

Health programs need to ensure that local health facilities are appropriately staffed and that the staff has the training, equipment, supplies and transport needed to support community–level work. For example, mobile health teams based at peripheral facilities need, at a minimum, steady supplies of vaccines and adequate transport. Additionally, compassionate and high–quality curative and referral care, including basic hospital and surgical care, lends credibility to the community–based work and the workers who provide it. Small, well–run first–level referral hospitals can be cost–effective in improving health and can serve as an important asset for the community to gain trust in the health system [59,60].

Health systems can benefit greatly from having a community–level worker implement evidence–based interventions in order to achieve high population coverage of these interventions. One recent analysis [61] concluded that almost two–thirds (59%) of maternal, prenatal, neonatal, and child deaths that could be prevented by all currently available interventions could be prevented with community–based approaches. Facility–based approaches would avert far fewer (20% at primary health care centers and 22% at hospitals).

Of course, the community–level workers who implement these interventions in collaboration with communities must be appropriately trained and supported; a recent Cochrane Review identified the need for adequate and standardized compensation or incentives for CHWs [62]. An effective strategy must be developed for promptly selecting and training new CHWs to replace those who are no longer functioning in this capacity. Although these decisions are normally made by program leaders in consultation with local communities, examples exist in which communities have taken full responsibility for this process [63]. In addition to continuing research on the capability of CHWs to provide specific interventions, more research will be needed on how many interventions a given CHW can take on and what training and supervision are required to maintain quality.

As we have seen in this analysis, empowering the community to be a partner with the health system can help strengthen community–based delivery strategies, as described in **Figure 1**. The finding supports the recent assertion of Marston et al. [64] that community participation (in which communities work together with health services for the co–production of health care) will be central for achieving the recently released World Health Organization global strategy for women’s and children’s health [65].

Community case management, routine systematic home visitation, participatory women’s groups, and outreach services provided by mobile teams represent important delivery strategies for improving MNCH in high–mortality, resource–constrained settings. These strategies are not the only approaches to implementing interventions that can improve child health, but they are the most common strategies used in the projects whose assessments are included in our database.

Routine systematic home visitation has the unique advantage of not only delivering key interventions to all who need them but also of ensuring that no one is left out. Marginalization and discrimination of sub–groups in high–mortality, resource–constrained settings are not uncommon, leading to many social barriers – in addition to geographic barriers – in accessing services at facilities or even at peripheral outreach points. Thus, for instance, home visits have proven to be an essential strategy for the final stages of polio eradication [66].

Cesar Victora, one of the widely acknowledged leaders of the global movement to improve MNCH, lamented that “We have the bullets [interventions] but not the guns [implementation strategies]” for a second child survival revolution [67]. The analysis provided here helps to point the way forward by identifying implementation strategies used by programs with demonstrated effectiveness.

### Study limitations

The word limits placed on peer–reviewed journal articles make it difficult to fully describe implementation strategies. Our data extraction process was set up to glean whatever information was available regarding these strategies. Our database has been strengthened by the inclusion of 116 assessments that are not peer–reviewed journal articles, and many of them describe their strategies in greater detail. Most of these additional 116 assessments are either unpublished evaluation reports that are publicly available or books. These documents are useful in part because they are not subject to the same space limitations as peer–reviewed articles and can provide more information. Further consolidation and analysis of the extensive and rich evidence about strategies for implementation of CBPHC projects described in the gray literature (including a rigorous examination of the quality of the assessments) would be useful but goes beyond the capacity of the current series of articles to address.

Another limitation of this study is that some of the findings reported here are based on subjective judgments of reviewers. However, the procedure we used – having each assessment reviewed independently by two researchers and then having a third resolve any differences – helps to mitigate this limitation.

A final limitation of our review is the overall difficulty of assessing community participation and engagement. While one of the strengths of our paper is highlighting and further describing the role of the community in implementing effective CBPHC projects, we also note that frameworks and indicators for assessing the quality and effectiveness of this critical dimension of CBPHC were rarely used in the assessments included in our review. Appropriate frameworks and indicators need to be used by future CBPHC projects so that they can more fully describe the role of the community in the process of implementation and better assess the contribution that this made to health outcomes. Useful and more robust approaches to describing and analyzing the process of community participation are available [68,69].

## CONCLUSIONS

This analysis provides an overview of the ways in which CBPHC projects have planned and evaluated their activities, how they collaborated with communities, how they have used CHWs, and how they have strengthened health systems. The evidence from this review supports the proposition that the application of these strategies can accelerate the decline in maternal, neonatal and child mortality in priority countries. These strategies require that the health system establish functional partnerships with community leaders and community members in order to achieve high levels of coverage of evidence–based interventions. Building the capacity of health systems to work with communities to implement these strategies is one of the priority tasks for ending preventable child and maternal deaths by 2030.

Using the strategies identified here for strengthening CBPHC to improve MNCH can establish an entry point for developing synergies with community–based approaches for the detection and treatment of HIV/AIDS [70], tuberculosis [71] and malaria [31] as well as for the promotion of family planning services [72], detection and treatment of adult non–communicable diseases [73], and the achievement of universal health coverage. This review supports the growing recognition that community–based programs in high–mortality, resource–constrained settings have a great potential for improving MNCH at low cost.

Nonetheless, awareness about the full potential of CBPHC is still not yet widespread, and evidence of the effectiveness of CBPHC at scale in priority settings remains limited. Determining the fit and feasibility, within existing local and health systems constraints, of CBPHC implementation strategies for MNCH interventions is a pressing challenge for national programs. Unleashing the full potential of communities as partners in the process of building effective health systems in high–mortality, resource–constrained settings is one of the great frontiers for global health in the 21^st^ century.
